# Biometals in rare neurodegenerative disorders of childhood

**DOI:** 10.3389/fnagi.2013.00014

**Published:** 2013-03-25

**Authors:** Sarah J. Parker, Jari Koistinaho, Anthony R. White, Katja M. Kanninen

**Affiliations:** ^1^Department of Neurobiology, A.I. Virtanen Institute for Molecular Sciences, University of Eastern FinlandKuopio, Finland; ^2^Department of Pathology, The University of MelbourneParkville, VIC, Australia

**Keywords:** metals, neurodegeneration, childhood, copper, iron, zinc

## Abstract

Copper, iron, and zinc are just three of the main biometals critical for correct functioning of the central nervous system (CNS). They have diverse roles in many functional processes including but not limited to enzyme catalysis, protein stabilization, and energy production. The range of metal concentrations within the body is tightly regulated and when the balance is perturbed, debilitating effects ensue. Homeostasis of brain biometals is mainly controlled by various metal transporters and metal sequestering proteins. The biological roles of biometals are vastly reviewed in the literature with a large focus on the connection to neurological conditions associated with ageing. Biometals are also implicated in a variety of debilitating inherited childhood disorders, some of which arise soon following birth or as the child progresses into early adulthood. This review acts to highlight what we know about biometals in childhood neurological disorders such as Wilson's disease (WD), Menkes disease (MD), neuronal ceroid lipofuscinoses (NCLs), and neurodegeneration with brain iron accumulation (NBIA). Also discussed are some of the animal models available to determine the pathological mechanisms in these childhood disorders, which we hope will aid in our understanding of the role of biometals in disease and in attaining possible therapeutics in the future.

## Introduction

The significance of biometals for the correct functioning of the human brain has long been discovered. The importance of metals such as iron, zinc, and copper results from the numerous roles that they have, such as stabilization of proteins and transcription factors, acting as co-factors for metallochaperones for cellular transport and their role in enzyme catalysis (Markossian and Kurganov, 2003; Butterworth, 2010). Deviations of metal homeostasis have been linked to neurodegenerative disorders such as Alzheimer's disease (AD), Parkinson's disease, Amyotrophic lateral sclerosis, and Huntington's disease (HD) (Dexter et al., 1993; Deibel et al., 1996; Jomova et al., 2010; Skjorringe et al., 2012). A risk factor in the development of many of these disorders appears to be an increase in age. Although the pathological characteristics of the disease are in most cases well-understood, the exact causes are often unknown.

Neurodegeneration is not restricted to an ageing population. Several debilitating neurodegenerative disorders affecting children have been identified. These include neurodegeneration with brain iron accumulation (NBIA), ATPase pathologies such as Wilson's/Menkes disease and neuronal ceroid lipofuscinoses (NCLs). These disorders share several pathological features with more common neurodegenerative diseases, including protein aggregation and oxidative stress. Aberrant biometal homeostasis has also been identified in children suffering from these diseases (Table 1). What is not clear is whether the distinct changes in metal levels cause neurodegeneration or occur as a result of the neurodegeneration. In some cases, the pathology associated with childhood disorders is clearly evident and often fatal at an early age. Changes in metal levels have also been identified in children with Autism spectrum disorder (ASD) which affects the behavior of children more so than hinder them physically. Dissecting the roles that biometals have in these disorders is fundamental for finding potential therapeutics to reduce or inhibit neuropathological changes associated with altered metal homeostasis (Yasuda et al., 2011).

**Table 1:** **Biometal alterations in childhood disorders**.

**Childhood disorder**	**Associated gene(s)**	**Biometal concentrations**
Wilson's disease	*ATP7B*	Copper ↑
Menkes disease	*ATP7A*	Copper ↓
Neuronal ceroid lipofuscinoses	At least 10 genes	Zinc, manganese, copper, iron, cobalt, sodium ↑
		Potassium, magnesium ↓
Neurodegenerative disorders with iron accumulation	At least 7 genes	Iron ↑

Since biometal homeostasis plays a major role in neurodegenerative disease, the aim of this review is to highlight the effects that metals have on the developing brain. Multiple childhood disorders arise from defects in the concentrations of metals within the body and brain but little is known about how these changes can be corrected or prevented to avoid or ameliorate disease. While most of the childhood disorders are inherited and disease progression can occur not long after birth, it is critical to determine the molecular disease characteristics and metal alterations within the brain and other tissues for the development of therapeutic treatments to provide these children with a chance at life.

## Wilson's disease

Wilson's disease (WD) is an autosomal recessive disease affecting copper metabolism (Bearn and Kunkel, 1954). Copper is involved in neurotransmitter synthesis, cellular respiration, scavenging of toxic free-radicals, and has an important role in maintaining homeostasis of other trace elements such as iron (Butterworth, 2010). However, in abundance, copper is toxic and thus strict regulation of levels is critical. Free copper ions are virtually non-existent in human blood (Linder and Hazegh-Azam, 1996) and are usually bound to a multitude of proteins and chaperones for transport around the body. WD presents in patients typically between the ages of 6–20 years, and has an estimated incidence of 1 in 30,000 (Reilly et al., 1993).

The defective gene involved in WD is ATPase Cu(2+) transporting beta polypeptide (*ATP7B*), which encodes for a membrane bound copper-transporting ATPase protein primarily located within the liver cells, as part of the trans golgi network (Cox and Moore, 2002). To date, approximately 300 mutations within *ATP7B* have been associated with WD (Merle et al., 2010). Normal copper metabolism involves the transfer of copper molecules to ceruloplasmin for transport into the plasma so it can circulate to other tissues or when in excess, to the bile for excretion (Terada et al., 1998, 1999). ATP7B is involved in transporting the copper molecules to ceruloplasmin. When ATP7B is non-functional, copper accumulates to toxic levels and severely damages the liver. The liver therefore releases copper directly into the bloodstream where copper continues to cause damage to other tissues, in particular the CNS. In the brain, accumulation of copper is most evident in the basal ganglia, the region which co-ordinates movement (DeLong et al., 1984), making WD primarily a movement disorder. This neuropathology caused by the build up of copper leads to patients suffering tremors of the arms or hands, unpredictable movement, difficulty with speaking, swallowing, and walking as well as confusion, dementia, and various psychological problems (Rosencrantz and Schilsky, 2011). Some cognitive deficits have also been noted (Middleton and Strick, 2000), and could arise due to disruptions of projections of the basal ganglia to the prefrontal cortex (Bolam et al., 2000). Gliosis is also observed in the brains of patients suffering from WD (Anzil et al., 1974). The mechanism for neurological damage caused by excess copper is still not fully understood. However, it is known that copper is involved in the production toxic free radicals (Samuele et al., 2005), and excess copper has been shown to inhibit Inhibitor of apoptosis proteins (IAPs) caused by toxic deposits of intracellular copper (Mufti et al., 2006).

WD is a progressive disorder and was ultimately a fatal disease until the discovery of the first treatment in 1951 (Denny-Brown and Porter, 1951). With the copper-chelating agent, dimercaptopropanol, the disease is now treatable (Denny-Brown and Porter, 1951). The most common treatment to date is the use of zinc salts, which block enteral copper absorption in the stomach. Various drugs are currently available for WD and ongoing compliance with treatment is necessary.

### Animal models of WD

The Long Evans Cinnamon (LEC) rat is one model used for *in vivo* studies of WD. Since showing symptoms of liver failure in 1987 (Yoshida et al., 1987), the rats were first used as a model for liver cancer and hepatitis. Upon further characterization, accumulation of hepatic copper and decreased ceruloplasmin activity were found. Years later when the gene responsible for WD was discovered, the rat homolog *atp7b* was found to be mutated in the LEC rats, with a significant deletion at the 3′ end of the gene (Wu et al., 1994). Although there are similarities in liver pathogenesis between the LEC rat and humans, this rodent model shows minimal evidence of neuropathology (Terada and Sugiyama, 1999). In contrast to the LEC rat model, ATP7B knockout mice exhibit both neuro- and liver pathology. In these mice, copper levels are dramatically low at birth with a gradual increase by 2 months of age, where hepatic copper levels are approximately 50 times higher compared to wild-type controls (Buiakova et al., 1999). Another interesting model for WD is the toxic milk mouse, which also has an autosomal recessive inheritance (Theophilos et al., 1996). Female mice with the *tx* milk mutation produce copper-deficient milk, which is fatal to their pups. The *tx* mice have a single nucleotide change in the *ATP7B* gene, and studies have shown this to cause changes in the ATP7B localization in the mammary gland, therefore leading to impaired copper transport and little to no copper given to the pups. The *tx* mothers retain this copper in the liver (Rauch, 1983; Michalczyk et al., 2000).

Both the LEC rat and *tx* milk mouse models arose from spontaneous mutations. There is only one genetically engineered mouse model for WD, the *ATP7b^−/−^* mouse, which was generated by Buiakova et al. (1999). *ATP7b^−/−^* mice are born copper-deficient and display neurological symptoms such as tremors and abnormal locomotor behavior. If nursed by a homozygous *ATP7b^−/−^*mother, the pups will die at around 2 weeks of age. However, if nursed by a heterozygous mother, copper concentrations begin to rise to toxic levels in the liver by the age of 6 weeks, irrespective of initial birth concentration. Similar to human disease, these mice accumulate copper in the liver due to impaired export of copper into the bile, making these mice an invaluable model for the study of WD (Lutsenko, 2008).

## Menkes disease

Menkes disease (MD) is an inherited disease related to copper metabolism, but in contrast to WD, it is characterized by a significant reduction in normal copper levels in the blood, liver, and brain (Danks et al., 1973). Disease progression is also more rapid than in WD (de Bie et al., 2007). The reduction of copper is due to decreased absorption of copper from the intestine and therefore reduced transfer to copper-requiring proteins and enzymes (Danks, 1988). Symptoms present within the first year of life, generally within 3 months of birth and without treatment, death is likely before the third year of life. MD is a rare childhood disease with an estimated worldwide incidence of 1 in 300,000 (Tonnesen et al., 1991), but can be higher in certain countries such as Australia (1 in 50,000 to 1 in 100,000) (Tumer and Moller, 2010).

MD has an X-linked mode of inheritance and is more prevalent in males than females (Kodama and Murata, 1999). At birth, babies appear normal, although premature labor has been documented (Uriu-Adams et al., 2010). An early sign in a baby with MD is the presence of sparse and abnormal hair, which looks fragile and twisted and appears to have a “kink” in it (Kaler, 2011). MD is thus also referred to as “Kinky Hair Syndrome.” The baby begins to exhibit normal cognitive behavior with smiling and normal childhood dialogue; however, these behaviors do not progress further. Babies do not exhibit increases in muscle tone and over time generally become weaker and spasticity of the limbs is observed. Toward the end of the babies' short life, they succumb to blindness and respiratory failure and premature death is often due to infection or the extreme neuronal degeneration observed. A less-severe form of MD is called occipital horn syndrome (OHS) which is also an X-linked recessive inherited disorder with mutations in *ATP7A*. These patients exhibit fewer neurologic abnormalities and have a longer lifespan (Tumer and Moller, 2010). Connective tissue abnormalities tend to be the focus of this disorder with calcification of the occipital bulb, leading to horn-like structures at the base of the brain.

This detrimental disease is caused by a mutation in the gene encoding the Copper transporting ATPase-1 (ATP7A) protein (Fatemi and Sarkar, 2002), which is also a “P-type” ATPase protein similar to ATP7B. To date, there are over 200 known mutations scattered throughout the gene (His and Cox, 2004). Although both ATP7A and ATP7B are involved in the transport of copper to secreted enzymes and metalloproteins, ATP7A is also involved in the absorption of intestinal copper and can transport copper to all cell types and vital structural enzymes (Barry et al., 2011). ATP7B is predominantly involved in the exit of copper from hepatocytes. These differences in protein function are demonstrated by the fact that MD patients exhibit a variable range of symptoms throughout the body as opposed to mainly liver damage seen in patients with WD. The neurological damage caused by mutant *ATP7A* is due to the decreased activity of the copper-dependent enzymes. Decreased cytochrome *c* oxidase leads to a reduction in ATP production by the mitochondria and causes apoptotic cell death (Borm et al., 2004). Dopamine-β-hydroxylase is necessary for neurotransmitter synthesis and its activity is decreased in response to low copper levels (Kaler et al., 1995). Superoxide dismutase 1 (SOD1) is a free-radical scavenger the activity of which has been linked to several neurodegenerative disorders, particularly ALS. Copper is a co-factor imperative for SOD1 activity. Levels of SOD1 are decreased in MD patients, contributing to lower protection against toxic oxidative species within the brain (Kaler, 1994). Collectively, the reduced activity of copper-dependent enzymes is thought to be responsible for the observed neurological damage in MD.

The cellular location of ATP7A is sensitive to the concentration of copper; when copper levels are low ATP7A remains on the membrane of the Golgi but when levels are increased, ATP7A is located to the plasma membrane where it can “pump” copper to other proteins and cuproenzymes (Hasan et al., 2012). In MD, where copper levels are low, it is essentially located to the Golgi and there is a deficiency of copper transport to essential enzymes, and other tissues.

The therapeutic benefit of injection of copper histidine as a copper replacement to humans has provided mixed results and is dependent on the mutation within the ATP7A gene (Kaler et al., 1995; Christodoulou et al., 1998). Some studies have shown that early administration of copper to babies can prevent the observed neurological decline but this is likely to be in patients with mutations in *ATP7A* that still allow for some transfer of copper to copper-requiring enzymes, but not severe mutations affecting initial absorption from the intestine (Tumer et al., 1996).

### Animal models of MD

Spontaneous mutations in the mouse homolog of *ATP7A*, *atp7a*, have been used for modeling defective ATP7A in humans (Kuo et al., 1997). These mice are called mottled mice and different mutations in the *atp7a* gene cause different neurological and connective tissue anomalies. The two mottled mice mutations mottled brindled (Mo^br^) and mottled macular (Mo^ml^) show severe reductions in copper within the liver (Hunt and Clarke, 1983), and die within 1–3 weeks of birth (Lenartowicz et al., 2012). This premature death can be prevented with injected copper within the first week of birth, as well as transgenic over expression of *atp7a* (Danks, 1986).

## Neuronal ceroid lipofuscinosis

The NCLs are a group of fatal autosomally-inherited neurodegenerative diseases occurring in childhood. The NCLs are also classified as lysosomal storage disorders since one of the main pathological characteristics is the accumulation of autofluorescent granules which contain lipids and proteins (lipopigments) in the lysosomes. Currently at least ten forms of NCLs have been described. Each form is distinguished by different defective genes (CLNs) and the age of onset (Cooper, 2003; Mitchison et al., 2004). Collectively, it is the most common neurodegenerative disorder of childhood with an estimated incidence of up to 1:12,500 (Rider and Rider, 1999). Although mutations in different genes cause different forms of the disease, all NCLs share common clinical symptoms; progressive loss of vision leading to blindness, motor and mental retardation, seizures, and finally premature death (Siintola et al., 2006). The exact function of these genes is still unknown (Tyynela et al., 2004). These genes encode soluble proteins and membrane-bound proteins, for example, CLN6 is a transmembrane protein which is localized to the endoplasmic reticulum (Heine et al., 2004). Children with defective CLN6 present with symptoms at the age of 3–5 years and tend to only live until 8–12 years of age. Currently there is no cure for any form of NCL (Sharp et al., 2003).

In NCLs, early reports have described some changes to metal levels, including iron and zinc, in blood cells of patients with NCL (Johansson et al., 1990). In a larger study of NCL patients, no association between “loosely bound” iron or copper levels in cerebrospinal fluid could be found (Heiskala et al., 1988). There is some evidence that altered metal homeostasis may also occur in other NCL models. A recent study reported changes in expression of the metal transporter, ZnT6, in both CLN3 and CLN6 mouse cerebellar neuron precursor cell lines (Cao et al., 2011). Furthermore, reduction in expression of the gene causing CLN7 NCL disease was observed in the brains of mice fed a high-iron diet (Johnstone and Milward, 2010), suggestive of a heightened sensitivity of NCL brains to fluxes in metal concentrations.

Recently, a link between brain biometal homeostasis and CLN6 disease was discovered. Sheep harboring a natural mutation in the CLN6 gene show significant increases in manganese and more so, zinc levels in all brain regions except the cerebellum and brain stem (Kanninen et al., 2013). In the CLN6 sheep model, the accumulation of biometals was evident at the symptomatic age of 12–14 months. Early studies on the metal contents of lipopigments from CLN6 sheep revealed no obvious association between metal contents and disease progression (Palmer et al., 1988). Metal contents were variable between lipopigments from different tissues in line with the normal role of lysosomes as storage depots for metals, indicating the lysosomal origin of the lipopigments. Changes in the metal contents of retina and retinal pigment epithelial cells of CLN6 sheep have been reported, and an association with lipopigment accumulation and altered manganese levels with increasing photoreceptor cell loss suggested (Jolly et al., 1989).

### Animal models of NCLs

To understand the pathogenesis of NCLs, gene-specific mutant mice have been engineered which contain mutations in *Cln1, Cln2, Cln3, Cln5, Cln7*, and *Cln10* genes (Cooper et al., 2006). Naturally occurring mutations in *Cln6* and *Cln8* have also been identified (Lonka et al., 2000; Gao et al., 2002). Many of these mouse models share phenotypic features in common with human patients such as neurological and motor deficits, seizures and neuronal loss, and as such, are valuable tools to study disease mechanisms and potential therapeutic approaches. Like the human form, the mouse models display variance in timing and sequence of events toward disease progression, highlighting the genetic differences between the NCLs (Cooper et al., 2006). As the function of the majority of these genes is still unknown, using these animal models will prove invaluable in elucidating the function and mechanism behind NCL pathologies. The naturally occurring mutation in the CLN6 gene of sheep mentioned above share a similar disease progression and phenotypic features similar to that of the human CLN6 NCL form (Jolly et al., 1989; Kay et al., 2011), making them an excellent model of NCL disease due to their complex CNS. However, with large animal models it may be challenging to obtain sufficient numbers of animals for assessment due to difficulties in breeding and housing such large animals.

## Neurodegenerative disorders with brain iron accumulation

As with copper and zinc mentioned above, levels of iron within the brain need to be tightly regulated. Iron homeostasis is regulated by proteins involved in absorption, transport, influx, and storage in tissues (Zhang and Enns, 2009). Increases in brain iron levels are typically associated with ageing and is highly prevalent in AD and HD. Neurodegenerative disorders with brain iron accumulation (NBIA) refers to a wide group of disorders affecting children, with similar outcomes: neurodegeneration and the accumulation of iron. The disease is caused by mutations in seven genes (Ke and Ming Qian, 2003). At birth, brain iron is not present, but levels rapidly increase until 20 years of age with an uneven distribution over time (Zorzi et al., 2012). The most recognized gene defects are mutations in pantothenate kinase (PANK2), which is an enzyme located within the mitochondria and regulates the formation of Coenzyme A, which is essential for deriving cellular energy via the tricarboxylic acid cycle (Leoni et al., 2012). PANK2 phosphorylates pantothenate and requires N-pantothenoyl-cysteine and pantetheine. The exact mechanism how mutations in *PANK2* lead to iron accumulation is not fully understood, but one hypothesis is that if cysteine cannot be utilized within the cell by PANK2, it accumulates over time. Cysteine can bind iron, and over time this may explain the observed accumulation of iron (Gregory and Hayflick, 2005). Although the mutation in *PANK2* directly does not cause neurodegeneration, it is the increased iron which contributes to the production of toxic and tissue-damaging reactive oxygen species (Ke and Ming Qian, 2003). Increases in iron have also been linked to several neurological disorders such as AD and PD (Batista-Nascimento et al., 2012). There are over 100 mutations in this gene and PANK2 disorders account for over half of all NBIA cases (Gregory and Hayflick, 2005). PANK2-specific NBIA, referred to as pantothenate kinase-associated neurodegeneration (PKAN) was formally called Hallervorden–Spatz syndrome before the name was changed to divert any association with the unethical euthanization of mentally ill patients during World War II by Dr. Hallervorden (Dooling et al., 1974). PKAN disease is autosomally inherited and presents in childhood as either the classical, early onset or atypical, late onset form (Hayflick et al., 2003). The extent of iron accumulation can be easily visualized and quantified using magnetic resonance imaging (MRI). The pattern exhibited by MRI-T2 weighted images shows the increased iron as “darkened spots” and provides a novel “eye of the tiger” image, making MRI an important diagnostic tool for NBIA (Schenck and Zimmerman, 2004).

The symptoms of classical PKAN disease become apparent within the first 10 years of life, with symptoms usually starting between 3 and 4 years of age. Affected children begin to have difficulty with walking and other movements, which is attributed to the build up of iron in the basal ganglia (Gregory and Hayflick, 2005), a region of the brain highly important in movement. Rigidity, spasticity, and speech difficulties soon follow (Dooling et al., 1974). Children become wheelchair bound within only a few years of diagnosis. Atypical PKAN disease begins later in life, with an average age of onset of 13–14 years and disease progression unfolds slowly over many years (Chan et al., 2008). Patients present firstly with behavioral problems such as depression and personality changes with movement and speech impediments following later. The symptoms of atypical PKAN can be highly variable from case to case.

Treatment for NBIA had been aimed at symptoms rather than the genetic defect causing the iron build up and severe neurodegeneration. Drugs aimed at reducing abnormal movement are widely prescribed, but to date, there is no preventative for NBIA disorders. Recently, reducing the accumulation of iron has been the target for treatment using iron-chelators, however this is met with varying efficacy (McNeill and Chinnery, 2011). Deferiprone is an iron chelator which acts to redistribute iron within the brain, by donating built-up iron to transferrin for transport elsewhere (Brittenham, 1992). The promising feature of this drug is the ability to cross the blood-brain barrier, necessary for targeting the build up of iron in CNS. However, no conclusive results can be drawn from these clinical studies due to the small number of patients trialed (Forni et al., 2008; Zorzi et al., 2011).

### Animal models of NBIA

Unlike the other childhood disorders discussed in this review, PKAN-NBIA does not have a suitable murine model of disease. A PANK2 knockout mouse was generated which showed retinal degeneration and growth retardation, but no defects on movement and gait were evident (Kuo et al., 2005; Brunetti et al., 2012). However, *Drosophila melanogaster* have been a powerful tool for understanding the biochemical basis of PANK2 mutations. Mutations in the homolog of PANK, *fumble (fbl*) exhibited a shortened lifespan, progressive locomotion defect, and degenerations in both central nervous system and retina (Gregory and Hayflick, 2005). Interestingly, mutations in PANK2 in both the *Drosophila* and mouse led to male sterility (Wu et al., 2009).

## Conclusions

The importance of metals in neurological disease has been outlined in numerous reviews over many decades, however, the exact mechanisms leading to aberrant metal homeostasis and why this is so detrimental to the brain remain poorly understood. While the brain is still developing during childhood, it is highly susceptible to slight changes in metal levels as outlined above with discussion of various childhood neurodegenerative disorders. Although certain therapeutics exist for some of these disorders, they mainly target disease symptoms and prolong the disease, rather than being preventative. It is the work forthcoming from *in vitro*, animal and clinical studies, which aim to add insight into the role of metal changes and consequences so potential therapeutics can be tested and hopefully one day applied to prevent disease progression.

### Conflict of interest statement

The authors declare that the research was conducted in the absence of any commercial or financial relationships that could be construed as a potential conflict of interest.
